# Sleeve Gastrectomy in a Patient With Left Hemidiaphragm Paralysis: A Case Report

**DOI:** 10.7759/cureus.27048

**Published:** 2022-07-20

**Authors:** Khalid M Alzahrani

**Affiliations:** 1 Surgery, Taif University, Taif, SAU

**Keywords:** preoperative assessment, body mass index, morbid obesity, laparoscopic sleeve gastrectomy, unilateral diaphragmatic paralysis

## Abstract

Unilateral diaphragmatic paralysis is a disorder in which one side of the diaphragm loses the capacity to contract to allow for adequate inspiration. The majority of patients suffering from unilateral diaphragmatic paralysis are asymptomatic and do not require treatment. The laparoscopic sleeve gastrectomy (LSG) is the most common bariatric surgical operation. In this case report, I present the case of a 36-year-old male with congenital left hemidiaphragm paralysis who had LSG for the treatment of morbid obesity. This case demonstrates the efficacy and feasibility of LSG in patients with asymptomatic hemidiaphragm paralysis. During surgery, surgeons must pay close attention to a variety of factors, including a stomach fundus that is situated high in a subcostal region with a narrow space to work.

## Introduction

The diaphragm is a muscular and fibrous tissue that is located between the thoracic and abdominal cavities [1]. It is the primary respiratory muscle in humans, and its weakness or paralysis can impair the fundamental activities of respiration [2].

The diaphragm begins to develop in the seventh week of gestation, and by the tenth week, it is fully formed. It is made up of three structures: the septum transverse, the right and left pleuroperitoneal membranes, and the esophageal dorsal mesentery. The diaphragmatic muscles are derived from the third to fifth cervical myotomes and are consequently supplied by the phrenic nerve, which arises from the third to fourth cervical roots [3]. Functionally, the diaphragm consists of two separate regions: the crural portion, which is mostly responsible for respiration, and the costal portion, which is primarily responsible for preventing gastroesophageal reflex [4].

When the diaphragm is weak, caudal displacement during inspiration is limited or nonexistent. This restricts the expansion of the thoracic cavity, leading to inadequate inspiration and hypoventilation. Because the diaphragm acts as two separate parts, the right and left hemidiaphragms, paralysis of both hemidiaphragms would result in substantial respiratory failure, whereas paresis of one hemidiaphragm may be completely asymptomatic [2]. Unilateral diaphragmatic paralysis is a disorder in which the right or left hemidiaphragm lacks the capacity to contract to allow for adequate inspiration. This can be caused by muscle problems in the diaphragm or lack of innervation of the hemidiaphragm by the phrenic nerve [5].

Laparoscopic sleeve gastrectomy (LSG) is the most frequently performed bariatric surgery, accounting for more than half of all primary bariatric procedures [6]. There are numerous published case series addressing concurrent hiatal hernia repair and LSG (LSG + HHR) [7,8], but very few case reports of LSG in patients with hemidiaphragm paralysis [9,10].

Herein, we report our experience to demonstrate the feasibility of performing LSG on a 36-year-old male presented with congenital asymptomatic hemidiaphragm paralysis and morbid obesity.

## Case presentation

A 36-year-old male with a history of congenital asymptomatic left hemidiaphragm paralysis since childhood with no previous trauma or surgical intervention presented to the Bariatric Surgery Clinic with a body mass index (BMI) of 47 kg/m^2^ (class 3 obesity). The weight was 148 kg and the height was 178 cm.

**Figure 1:**
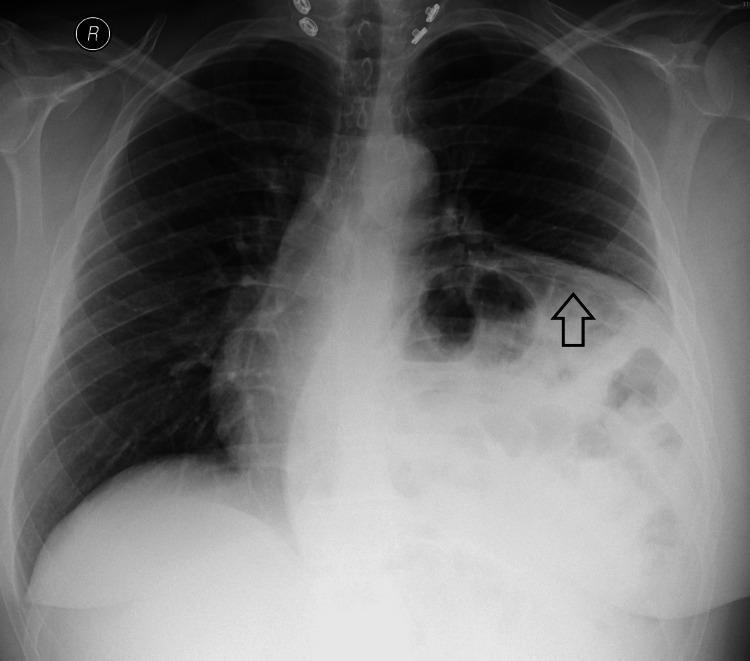
Chest x-ray showing elevated left hemidiaphragm.

The patient was assessed by a multidisciplinary team, including a bariatric surgeon, pulmonologist, and anesthesiologist. Preoperative assessment includes a complete physical exam, complete blood count (CBC), electrolytes, liver function test (LFT), renal function test (RFT), thyroid function test (TFTs), vitamin D and vitamin B12 levels, chest x-ray (CXR), electrocardiogram (ECG), esophagogastroduodenoscopy (EGD), and pulmonary function test (PFT). The patient’s preoperative laboratory investigations were all within the normal limit. CXR showed a left hemidiaphragm that was higher than the right one with a more acute costophrenic angle (Figure 1). EGD was normal with no hiatal hernia; ECG and PFT were normal. 

The LSG was performed with the patient in a reverse Trendelenburg position and split-leg supine while the surgeon was standing between the legs. A Veress needle (Fortala Laboratories Shenzhen Limited, Shenzhen, China) was inserted into the palmer's point to start the pneumoperitoneum, and it was maintained at 15 mm Hg of intra-abdominal pressure. A 12-mm optical trocar was inserted 15 cm below the xiphoid in the upper abdomen. At the left upper quadrant, another 12-mm trocar was inserted. The other three 5-mm trocars were inserted at the right upper quadrant, the left subcostal anterior axillary line, and finally at the sub-xiphoid area for the liver retractor.

The gastric fundus, greater curvature, and spleen were found above the left costal margin, just below the elevated left hemidiaphragm, but the esophagogastric junction (EGJ) was in a normal position. The dissection of the omentum from the greater curvature was started 4 cm proximal to the pyloric sphincter using Maryland-style jaws LigaSure™ device (5 mm-37 cm) (Medtronic Parkway, Minneapolis, MN, USA) and was continuing proximally into the angle of his. Complete dissection and mobilization of the fundus by division of the short gastric vessels were difficult due to the very high-located spleen in a confined subcostal space. A calibrating bougie of 36 Fr size was inserted in the stomach, then the gastric tubularization was done using the Endo GIA™ ultra universal stapler (Medtronic Parkway, Minneapolis, MN, USA) with four purple and one gold 60 mm staple load. Bleeding from the staple line was controlled using 5 mm clips.

No intraoperative or postoperative complications were reported. The patient was started with the clear liquid diet in the early morning on the postoperative day one and he was discharged on the same day. He was followed up in the clinic one, three, six, nine, and 12 months postoperatively with no active complaints. His weight at one-year post-LSG was 91 kg with a BMI of 28.7 kg/m^2^.

## Discussion

Diaphragmatic paralysis is a rare condition that affects less than 0.05% of the population and is more common in males [11]. The vast majority of patients with unilateral diaphragmatic paralysis are asymptomatic and do not require any treatment. Diaphragmatic plication can be performed if the weakness persists for over a year [2]. Nevertheless, patients with morbid obesity and progressive neuromuscular disease should not undergo this procedure [12,13].

Due to the uncommon occurrence of concurrent morbid obesity and unilateral diaphragmatic paralysis, few studies on LSG in patients with hemidiaphragmatic paralysis have been published. This case study supports the previous report describing the efficacy and feasibility of LSGs in patients with hemidiaphragmatic paralysis [9,10]. In a previous study by Kelso et al., they reported that sleeve gastrectomy aids in the recovery of hemidiaphragm paralysis caused by iatrogenic phrenic nerve injury [10]. While Hayes et al. described the simultaneous performance of laparoscopic sleeve gastrectomy and plication of the left hemidiaphragm in a morbidly obese patient with an iatrogenic paralyzed left hemidiaphragm [9]. In contrast to this case report, the hemidiaphragmatic paralysis in the previous reports was due to iatrogenic causes after cardiac interventions [9,10].

Various studies have found that exercise tolerance is drastically reduced in patients with hemidiaphragm paralysis and obesity [14,15]. Therefore, pulmonary function tests and anesthesia evaluations are required prior to surgical intervention. The anesthesiologist must be able to manage increased respiratory secretions, respiratory infections, and prolonged intubation. During surgery, surgeons must pay close attention to a number of factors, including the fundus of the stomach, spleen, and short gastric vessels that are highly located in a subcostal region with a narrow space to work. Therefore, complete dissection and mobilization of the fundus by division of the short gastric vessels are challenging and need to be done carefully and meticulously to avoid bleeding from short gastric vessels or spleen and to prevent spleen infarction. Moreover, these patients must be operated on and treated by an expert bariatric surgeon, as they may need conversion to open surgery, identification of altered anatomy, repair of unanticipated intraoperative injuries, or treatment of postoperative complications.

## Conclusions

Unilateral diaphragmatic paralysis is a rare condition, as is its co-occurrence with morbid obesity. Patients with morbid obesity undergoing laparoscopic sleeve gastrectomy with concomitant unilateral diaphragmatic paralysis should receive a comprehensive preoperative evaluation consisting of a PFT, EGD, CXR, and ECG. After evaluation by a bariatric multidisciplinary team, laparoscopic sleeve gastrectomy is safe and effective for patients with asymptomatic unilateral diaphragmatic paralysis.

Management can be difficult for both the anesthesiologist and the surgeon; therefore, it is advisable to leave it to a professional bariatric surgeon at a tertiary bariatric hospital with adequate facilities.
